# *Drosophila* Cyclin G and epigenetic maintenance of gene expression during development

**DOI:** 10.1186/s13072-015-0008-6

**Published:** 2015-05-07

**Authors:** Camille A Dupont, Delphine Dardalhon-Cuménal, Michael Kyba, Hugh W Brock, Neel B Randsholt, Frédérique Peronnet

**Affiliations:** Sorbonne Universités, UPMC Univ Paris 06, Institut de Biologie Paris-Seine (IBPS), UMR 7622, Developmental Biology, 9, quai Saint-Bernard, F-75005 Paris, France; CNRS, IBPS, UMR 7622, Developmental Biology, 9, quai Saint-Bernard, F-75005 Paris, France; Lillehei Heart Institute and Department of Pediatrics, University of Minnesota, 2231 6th Street SE, Minneapolis, MN 55455 USA; Department of Zoology, University of British Columbia, 6270 University Boulevard, V6T 1Z4 Vancouver, BC Canada

**Keywords:** Polycomb, Trithorax, Cyclin G, Homeotic

## Abstract

**Background:**

Cyclins and cyclin-dependent kinases (CDKs) are essential for cell cycle regulation and are functionally associated with proteins involved in epigenetic maintenance of transcriptional patterns in various developmental or cellular contexts. Epigenetic maintenance of transcription patterns, notably of *Hox* genes, requires the conserved *Polycomb*-group (PcG), *Trithorax*-group (TrxG), and *Enhancer of Trithorax and Polycomb* (ETP) proteins, particularly well studied in *Drosophila*. These proteins form large multimeric complexes that bind chromatin and appose or recognize histone post-translational modifications. *PcG* genes act as repressors, counteracted by *trxG* genes that maintain gene activation, while *ETPs* interact with both, behaving alternatively as repressors or activators. *Drosophila* Cyclin G negatively regulates cell growth and cell cycle progression, binds and co-localizes with the ETP Corto on chromatin, and participates with Corto in *Abdominal*-*B Hox* gene regulation. Here, we address further implications of Cyclin G in epigenetic maintenance of gene expression.

**Results:**

We show that Cyclin G physically interacts and extensively co-localizes on chromatin with the conserved ETP Additional sex combs (ASX), belonging to the repressive PR-DUB complex that participates in H2A deubiquitination and *Hox* gene silencing. Furthermore, Cyclin G mainly co-localizes with RNA polymerase II phosphorylated on serine 2 that is specific to productive transcription. *CycG* interacts with *Asx*, *PcG*, and *trxG* genes in *Hox* gene maintenance, and behaves as a *PcG* gene. These interactions correlate with modified ectopic Hox protein domains in imaginal discs, consistent with a role for Cyclin G in *PcG*-mediated *Hox* gene repression.

**Conclusions:**

We show here that *Drosophila CycG* is a Polycomb-group gene enhancer, acting in epigenetic maintenance of the *Hox* genes *Sex combs reduced* (*Scr*) and *Ultrabithorax* (*Ubx*). However, our data suggest that Cyclin G acts alternatively as a transcriptional activator or repressor depending on the developmental stage, the tissue or the target gene. Interestingly, since Cyclin G interacts with several CDKs, Cyclin G binding to the ETPs ASX or Corto suggests that their activity could depend on Cyclin G-mediated phosphorylation. We discuss whether Cyclin G fine-tunes transcription by controlling H2A ubiquitination and transcriptional elongation *via* interaction with the ASX subunit of PR-DUB.

**Electronic supplementary material:**

The online version of this article (doi:10.1186/s13072-015-0008-6) contains supplementary material, which is available to authorized users.

## Background

Cyclins and cyclin-dependent kinases (CDKs) are classically described as essential regulators of the cell cycle. However, the discovery of new cyclins, along with extensive studies of cell cycle regulators, have revealed that they are involved in diverse processes ranging from metabolism to stem cell self-renewal, and play key roles in regulation of transcription [1,2]. Indeed, cyclin/CDK complexes orchestrate the transcription cycle by dynamically phosphorylating the heptapeptide repeats which form the C-terminal domain (CTD) of the largest RNA polymerase II (RNA Pol II) subunit [3,4]. Notably, transcriptional elongation requires phosphorylation of the CTD on serine 2 by the positive transcription elongation factor b (P-TEFb), formed by CDK9 and Cyclin T or K [5-7].

Cyclins and CDKs can also directly alter gene expression. For example, mammalian Cyclin D1 binds several core transcription factors in regions close to transcription start sites [8], thus participating in regulation of adjacent genes. Furthermore, a cyclin/CDK complex determines the activity of the multi-subunit complex mediator that plays a fundamental role in eukaryotic gene regulation. Indeed, the mediator kinase subunit, composed of Cyclin C/CDK8, MED12, and MED13 in *Drosophila*, promotes either transcriptional activation or repression depending on the context [9-12]. Interestingly, MED12 and MED13, encoded by *kohtalo* (*kto*) and *skuld* (*skd*), were initially identified as suppressors of homeotic phenotypes induced by mutation of *Polycomb* (*Pc*), an epigenetic repressor of transcription [13]. However, *kto* and *skd* are also involved in epigenetic repression of the *Hox* gene *Ubx* during development [14]. These data link the mediator kinase subunit to the epigenetic mechanisms that ensure faithful transmission of chromatin states from mother to daughter cells.

During development, epigenetic maintenance of gene expression patterns is under control of evolutionary conserved proteins encoded by the *Polycomb*-*group* (*PcG*), *trithorax*-*group* (*trxG*), and *Enhancer of Trithorax and Polycomb* (*ETP*) genes, whose roles have been particularly well studied for homeotic (*Hox*) gene regulation in *Drosophila* [15-18]. *PcG* genes are involved in long-term gene repression, whereas *trxG* genes participate in maintenance of gene activation and counteract *PcG* action. *ETP* genes have been mainly characterized in *Drosophila*. They interact with both *trxG* and *PcG* genes, and behave alternatively as repressors or activators of target genes [17-19].

The Trithorax-group (TrxG) and PcG proteins form large multimeric complexes that bind chromatin and appose or recognize histone post-translational modifications. TrxG complexes are mostly involved in gene activation (for a review see [20]). They comprise histone modifying complexes, such as trithorax activating complex 1 (TAC1) containing the SET-domain histone methyltransferase TRX that trimethylates lysine 4 of histone H3 (H3K4me3) [21], or the ASH1 complex containing the histone methyltransferase absent, small, or homeotic discs 1 (ASH1), which methylates histone H3 and histone H4 [22-24]. TrxG complexes also include ATP-dependent chromatin remodeling complexes, such as BAP that contains the ATPase Brahma (BRM) [25]. On the contrary, PcG complexes are involved in epigenetic maintenance of gene silencing (for a review see [15]). The conserved polycomb repressive complex 2 (PRC2) contains several PcG proteins, including extra sex combs (ESC) and enhancer of zeste [E(Z)], a SET-domain histone methyltransferase that trimethylates histone 3 on lysine 27 (H3K27me3). A second repressive complex, PRC1, comprises the PcG proteins polycomb (PC) and polyhomeotic (PH). PRC1 silences genes through ubiquitination of histone H2A on lysine 119 and chromatin compaction [26,27]. Other PcG complexes include the recently identified polycomb repressive deubiquitinase (PR-DUB) complex consisting of the deubiquitinase Calypso and the ETP Additional sex comb (ASX) in *Drosophila* [28]. Mammalian PR-DUB contains BAP1, homolog to Calypso, ASXL1 and ASXL2, two ASX homologs, as well as several additional partners [29,30]. PR-DUB catalyzes deubiquitination of H2AK119, binds PcG targets and is essential for promoter silencing [28-31]. However, *Drosophila* ASX, as well as its murine homologs, are required for both activation and repression of *Hox* genes, which makes them genuine ETPs [32-34].

ETPs also comprise the evolutionary conserved GAGA factor that interacts with both BAP and PRC complexes in the regulation of *Hox* genes [35] and the proteins Corto and dorsal switch protein 1 (DSP1) in *Drosophila* [36,37]. Corto interacts physically with GAGA and DSP1 which are both involved in the recruitment of PcG complexes to Polycomb Response Elements (PRE) [38]. As several ETPs are differently recruited to PREs depending on tissues or developmental stages, it has been proposed that different combinations of ETPs could favor the recruitment of either PcG or TrxG complexes, thus participating in maintenance of silenced or active states [37].

*Drosophila* Cyclin G was first isolated during a two-hybrid screen using the ETP Corto as bait [39]. Cyclin G has two mammalian homologs, Cyclin G1 and G2, whose functions remain elusive. *CCNG1* that encodes Cyclin G1 is a direct target of the tumor-suppressor p53 [40]. Its induction following γ-irradiation leads to cell cycle arrest at the G2/M transition, thus allowing DNA damage repair [41]. On the contrary, overexpression of *CCNG1* activates the proliferation of colon carcinoma cells [42]. Overexpression of *CCNG2* that encodes Cyclin G2 induces cell cycle arrest at the G1/S transition [43]. Like Cyclin G2 [44], *Drosophila* Cyclin G bears a PEST sequence at its C-terminal extremity. Furthermore, Cyclin G negatively regulates both cell growth and cell cycle progression, preventing G1/S transition and slowing down the S phase [45,46]. Collectively, these characteristics suggest that *Drosophila* Cyclin G behaves more like Cyclin G2. Cyclin G co-localizes at many sites with the ETP Corto as well as with the PcG protein PH on larval polytene chromosomes, suggesting that it is involved in the control of gene expression [39]. Although a direct role of mammalian G-type cyclins in gene expression has not been reported, overexpression of *CCNG1* in human cells induces chromatin relaxation [47]. Genetic interactions between *CycG* (encoding *Drosophila* Cyclin G) and *corto* showed that both genes are involved in regulating expression of the homeotic gene *Abdominal*-*B* (*Abd*-*B*) in the early pupal epithelium, *corto* acting as a repressor and *CycG* as an activator of *Abd*-*B* [39]. Furthermore, Cyclin G and Corto bind the *iab*-*7 cis*-regulatory element as well as the promoter of *Abd*-*B* in embryos [39]. Altogether, these data strongly suggest a role for Cyclin G in regulation of *Hox* gene expression during development.

In this work, we address the involvement of Cyclin G in epigenetic maintenance of *Hox* gene expression in *Drosophila*. We first demonstrate that Cyclin G also interacts with the ETP ASX, and extensively co-localizes with ASX on polytene chromosomes. We next show that *CycG* genetically interacts with *Asx* in maintenance of *Hox* genes. Interestingly, *CycG* also interacts with several *PcG* and *trxG* genes and behaves genetically as a *PcG*, since loss of *CycG* enhances *PcG*-mediated homeotic phenotypes and suppresses *trxG*-mediated ones, whereas overexpression of *CycG* has the opposite effect. These genetic interactions were correlated with modifications of ectopic Hox protein domains in imaginal discs, suggesting a role for Cyclin G in *PcG*-mediated *Hox* gene repression during development. Surprisingly, we found that Cyclin G largely co-localizes with C-terminal domain of RNA polymerase II (RNA Pol II CTD) phosphorylated on serine 2, suggesting a role in productive transcription. We propose that Cyclin G acts as an activator or a repressor of transcription depending on the developmental stage, the tissue, or the target gene.

## Results

### *Drosophila* Cyclin G interacts with the enhancer of Trithorax and Polycomb ASX

Cyclin G physically interacts with the ETP Corto *in vivo*, and the Cyclin G N-terminal domain (amino-acids 1 to 130) is necessary and sufficient for this interaction [39]. Interestingly, a yeast two-hybrid screen to isolate interactors of the ETP ASX also identified Cyclin G as a potential partner. In this screen, the ASX-C terminal domain (ASX-C, amino-acids 1139 to 1668), containing a PHD domain (amino-acids 1634 to 1665), was used as bait against a two-hybrid library of 0 to 12 h embryonic complementary DNAs (cDNAs) [48]. A deleted form of Cyclin G containing only the 275 N-terminal residues interacted strongly with ASX-C in two-hybrid assays (data not shown). These results showed that Cyclin G residues 1 to 275, N-terminal to the cyclin box, were sufficient for the interaction with ASX.

**Figure 1:**
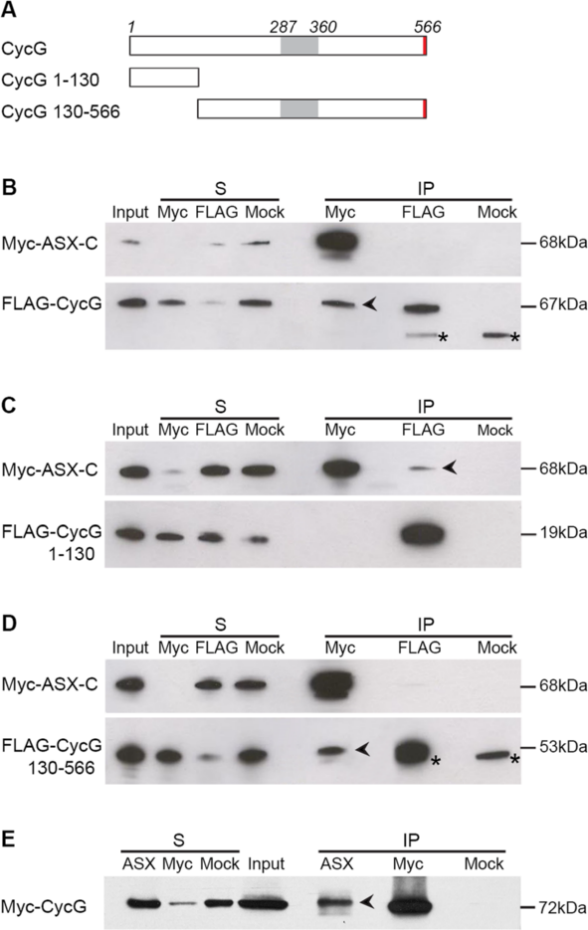
Cyclin G physically interacts with ASX *in vivo*. **(A)** Schematic representation of Cyclin G full-length (CycG) and truncated forms (CycG 1 to 130 and CycG 130 to 566 corresponding to amino-acids 1 to 130 and 130 to 566, respectively). Grey box: Cyclin domain; red box: PEST sequence. **(B**-**D)** Cyclin G interacts with ASX-C (amino-acids 1139 to 1668) in S2 cells; black arrowheads indicate co-immunoprecipitation. FLAG-CycG **(B)** and FLAG-CycG 130–566 **(D)** co-immunoprecipitate with Myc-ASX-C. Note that FLAG-CycG 130 to 566 co-migrates with IgG heavy chains. **(C)** Myc-ASX-C co-immunoprecipitates with FLAG-CycG 1 to 130. Immunoprecipitations were performed with anti-Myc, anti-FLAG, or anti-HA (Mock) antibodies. Immunoprecipitated proteins were revealed by Western blot, using anti-Myc (top panel) or anti-FLAG antibodies (bottom panel). In **(B)** and **(D)**, asterisks indicate IgG heavy chains. S: supernatant after immunoprecipitation; IP: protein G-agarose beads. Five percent of the input or supernatant and 50% of the immunoprecipitate were loaded onto the gels. **(E)** Myc-Cyclin G co-immunoprecipitates with endogenous ASX in *da* > *Myc*-*CycG* ^*ΔP*^ third instar larvae. Immunoprecipitated proteins were revealed by Western blot, using anti-Myc antibody.

To further characterize the interaction between Cyclin G and ASX, we co-transfected S2 cells with *pAct*::*Myc*-*AsxC* and *pAct*::*FLAG*-*CycG* and performed immunoprecipitation using anti-Myc and anti-FLAG antibodies. FLAG-Cyclin G co-immunoprecipitated with Myc-ASX-C (Figure 1B), confirming the interaction between Cyclin G and ASX. To determine which Cyclin G region mediates this interaction, we next constructed different vectors expressing FLAG-tagged, truncated forms of Cyclin G (Figure 1A). Myc-ASX-C co-immunoprecipitated with the 130 N-terminal residues of Cyclin G (Figure 1C). Hence this domain, which also mediates the interaction with Corto, was sufficient to bind ASX. However, Cyclin G deleted of this N-terminal domain also co-immunoprecipitated with ASX-C (Figure 1D), indicating that other parts of Cyclin G interact with ASX-C.

To test the interaction between Cyclin G and ASX in more native conditions, we performed immunoprecipitation using protein extracts of third instar larvae overexpressing a Myc-tagged form of Cyclin G (*da* > *Myc*-*CycG*^*ΔP*^) using anti-Myc and anti-ASX antibodies. Myc-Cyclin G co-immunoprecipitated with ASX (Figure 1E), demonstrating that Cyclin G interacts with endogenous ASX *in vivo*.

Taken together, these results confirm that Cyclin G interacts with ASX, and show that the Corto-interacting domain, that is, amino-acids 1 to 130 of Cyclin G, is sufficient for this interaction.

### Cyclin G mainly localizes on active chromatin

**Figure 2:**
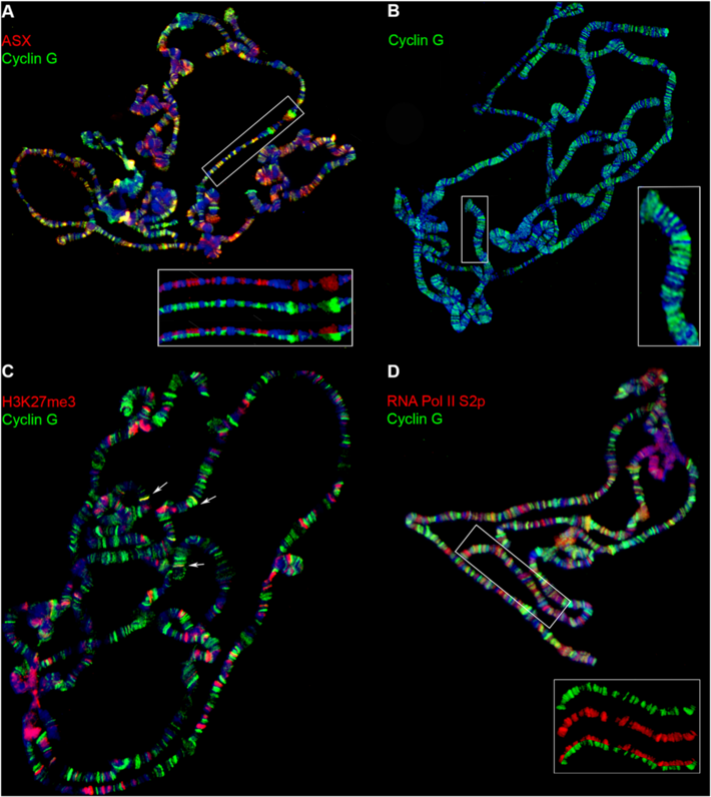
Cyclin G co-localizes with ASX and binds mainly active chromatin. Immunostaining of polytene chromosomes from *w* ^*1118*^ third instar larvae. DNA was stained with DAPI (blue). **(A)** Cyclin G (green) and ASX (red) co-localize at many sites. Bottom: close-up view of the region framed by a white rectangle. **(B)** Cyclin G (green) binds DAPI interbands. Right: close-up view of the region framed by a white rectangle. **(C)** Cyclin G (green) and H3K27me3 (red) are almost completely exclusive. Rare co-localizations are shown with white arrows. **(D)** Cyclin G (green) co-localizes at many sites with RNA Pol II phosphorylated on serine 2 (red). Bottom: close-up view of the region framed by a white rectangle.

Since Cyclin G physically interacts with two ETPs (Corto and ASX), and since we previously established that Cyclin G co-localizes with Corto at many loci on polytene chromosomes of larval salivary glands [39], we tested whether Cyclin G also shares binding sites with ASX on chromatin. We co-immunostained salivary gland polytene chromosomes of wild-type larvae with anti-Cyclin G and anti-ASX antibodies. We detected an important overlap of Cyclin G and ASX binding sites (Figure 2A), indicating that interaction of Cyclin G with ASX could take place on chromatin.

Furthermore, Cyclin G preferentially localized at DAPI interbands (Figure 2B), suggesting that this protein mainly binds open chromatin. This observation led us to test whether Cyclin G binding was preferentially coupled with an active or a repressed chromatin state, by examining its co-localization with specific marks on polytene chromosomes. Comparison of Cyclin G binding sites with the distribution of the repressive histone mark H3K27me3 showed that very few Cyclin G-bound loci were positive for H3K27me3 (Figure 2C). Conversely, Cyclin G co-localized largely with RNA Pol II phosphorylated on serine 2 (Figure 2D). These results suggest that Cyclin G binds mainly open chromatin and might be associated with genes undergoing transcription.

### *CycG* genetically interacts with *Asx*

The physical interaction between Cyclin G and ASX as well as their extensive co-localization on chromatin led us to examine genetic interactions between *CycG* and *Asx*, using two loss of function alleles of *Asx*, *Asx*^*XF23*^, and *Asx*^*3*^ [49,50]. To address the effects of *CycG* misregulation on *Asx* phenotypes, we combined these *Asx* mutations with *CycG* loss of function [ubiquitous inactivation by RNA interference using the *UAS*::*dsCycG2* transgene driven by *da*::*Gal4* (*da* > *CycG RNAi*)] or gain of function [ubiquitous overexpression of *CycG* using the *UAS*::*CycG*^*ΔP*^ transgene (*da* > *CycG*^*ΔP*^ encoding Cyclin G deleted of amino-acids 542–566)]. We focused on the effect of *CycG* misregulation on *PcG*-like homeotic transformations induced by *Asx* mutations. Importantly, these homeotic phenotypes were never detected in *CycG* loss of function or gain of function flies. All genetic interaction data are shown in Additional file 1.

**Figure 3:**
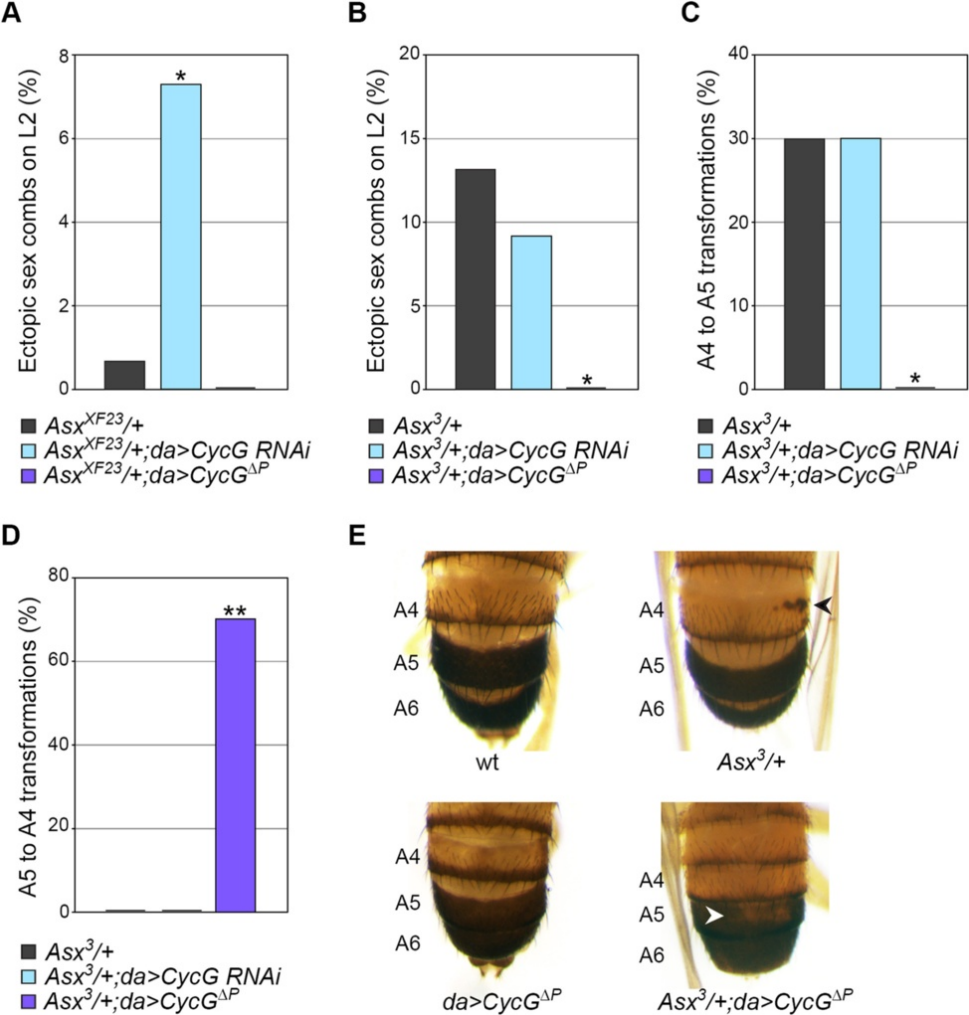
*CycG* genetically interacts with *Asx*. **(A**, **B)** Effect of *CycG* misregulation on the penetrance of ectopic sex combs on L2 induced by **(A)** *Asx* ^*XF23*^ and **(B)** *Asx* ^*3*^. **(C**, **D)** Penetrance of A4 to A5 **(C)** and A5 to A4 **(D)** abdominal segment transformations in *Asx* ^*3*^/+, *Asx* ^*3*^/+;*da* > *CycG RNAi* and *Asx* ^*3*^/+;*da* > *CycG* ^*ΔP*^/+ males. **(E)** Posterior abdomens of adult males showing wild-type male pigmentation in A4 to A6, A4 to A5 transformation in *Asx* ^*3*^/+ male (black arrowhead), and A5 to A4 transformation in *Asx* ^*3*^/+;*da* > *CycG* ^*ΔP*^/+ male (white arrowhead). Fisher’s exact test, *P < 0.01 **P < 0.0001 (*n* ≥ 30). L2: mesothoracic leg. A4, A5, A6: abdominal segments 4, 5, and 6.

*PcG* genes derived their name from the most conspicuous common phenotype of adult males carrying *PcG* mutations: ectopic sex combs on posterior legs. Indeed, in *Drosophila melanogaster*, this male-specific structure, composed of specialized bristles called teeth, is specific to the first tarsal segment of prothoracic legs (L1). The occurrence of sex comb teeth on mesothoracic (L2) and metathoracic legs (L3) thus indicates a partial transformation of L2 and L3 into L1. Males heterozygous for *Asx*^*XF23*^ presented ectopic sex combs on L2 with a lower penetrance than *Asx*^*3*^ heterozygous males. *CycG* loss of function significantly enhanced penetrance of L2 sex combs induced by *Asx*^*XF23*^ (Figure 3A) but did not modify that of *Asx*^*3*^ (Figure 3B). Furthermore, *CycG* gain of function significantly suppressed L2 sex combs induced by *Asx*^*3*^ (Figure 3B). To summarize, *CycG* gain of function opposed an *Asx*-induced *PcG* phenotype on L2, whereas *CycG* loss of function enhanced this phenotype. Therefore, *CycG* behaved as an enhancer of *Asx* regarding homeotic transformation of L2 into L1. This suggests that *CycG* participates with *Asx* in the maintenance of mesothoracic leg identity.

Maintenance of abdominal segment identity also relies on *PcG*, *trxG*, and *ETP* genes. In wild-type *Drosophila* males, tergites of the fifth (A5) and sixth (A6) abdominal segments present a uniform dark pigmentation, whereas tergites of more anterior abdominal segments show only a posterior stripe of dark pigmentation. Thirty percent of *Asx*^*3*^/+ males presented darkly pigmented patches on the anterior part of the fourth abdominal tergite (A4), indicating a partial transformation of A4 into a more posterior segment, a phenotype observed in some *PcG* mutants (Figure 3E). As shown on Figure 3C, this phenotype was not affected by *CycG* loss of function, but was completely suppressed by *CycG* gain of function. Interestingly, 70% of *Asx*^*3*^/+; *da* > *CycG*^*ΔP*^ males presented small patches lacking dark pigmentation on tergite A5 (Figure 3D,E). This corresponds to a partial transformation of A5 into segment A4, which is a classical *trxG* homeotic transformation. Hence, in the abdomen, *CycG* gain of function not only suppressed the typical *PcG* phenotype induced by *Asx*^*3*^ but also shifted it toward a *trxG* phenotype.

Together, these results reveal that *CycG* and *Asx* are engaged in complex genetic interactions and suggest a role for *CycG* similar to that of a *PcG* gene in maintenance of segmental identities during development.

### *CycG* loss of function enhances *PcG*-induced homeotic transformations

The genetic interactions reported above, the physical interaction between Cyclin G and the ETPs ASX and Corto, and the co-localization on chromatin of Cyclin G with ASX as well as with Corto and the PcG protein PH, led us to further examine genetic interactions between *CycG* and *PcG*, *trxG*, or *ETP* genes. We associated *CycG* misregulation with alleles of these genes (listed in Additional file 2) reported to induce a visible dose-sensitive phenotype, alone or in combination with other *PcG*, *trxG*, or *ETP* mutations. All genetic interaction data are shown in Additional file 1.

**Figure 4:**
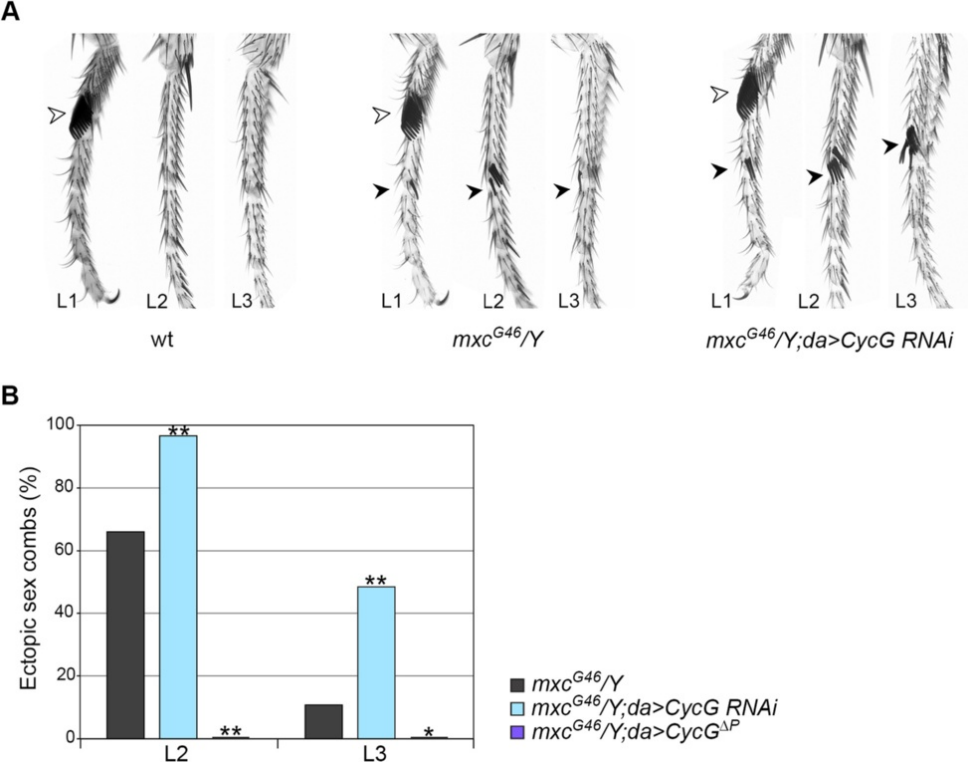
*CycG* misregulation alters ectopic sex comb phenotypes of an *mxc* mutant. **(A)** Expressivity of mesothoracic leg (L2) and metathoracic leg (L3) transformations into prothoracic leg (L1). The wild-type sex comb is marked by a white arrowhead. *mxc* ^*G46*^/Y males present ectopic sex comb teeth on distal L1, on L2 and L3 (black arrowheads). Only phenotypes affecting L2 and L3 were rated. These phenotypes are enhanced by *CycG* loss of function. **(B)** Penetrance of the ectopic sex combs phenotype on male legs. *CycG* loss of function enhances *mxc* ^*G46*^ induced ectopic sex combs on L2 and L3, whereas *CycG* gain of function suppresses ectopic sex combs on both legs. Fisher’s exact test, *P < 0.05 **P < 0.0001 (*n* ≥ 50).

Contrary to *Asx* mutations, mutant alleles of the ETPs *corto* and *Dsp1* induced no *PcG*-like leg transformation, whether alone or in combination with *CycG* misregulation. We next analyzed interactions with *mxc* (multi sex combs) and *crm* (cramped), genetically classified as *PcG* genes [51,52], although their products have not been found in PcG complexes so far. *mxc*^*G46*^/Y males present sex comb teeth on posterior legs [52], indicating a partial transformation into L1 (Figure 4A). Indeed, 66% of L2 and 11% of L3 (*n* = 56) carried at least one sex comb tooth (1.4 ± 1.3 teeth on L2 and 0.2 ± 0.5 on L3). Pharate *mxc*^*G46*^/Y males presented similar phenotypes (Additional file 1). As both gain and loss of function of *CycG* induced *mxc*^*G46*^ male lethality just prior to adult emergence, ectopic sex combs were scored in pharates. *CycG* loss of function enhanced the expressivity of this phenotype (3.7 ± 2.3 teeth on L2 and 1.3 ± 2.0 on L3, *n* ≥ 56; *t*-test, *P* < 0.0001) (Figure 4A). Penetrance of the phenotype was also enhanced by *CycG* loss of function (Figure 4B). Conversely, *CycG* gain of function completely suppressed L2 and L3 sex combs of *mxc*^*G46*^/Y males (Figure 4B). *CycG* misregulation *mxc*^*G46*^ males presented no other phenotypes than modified ectopic extra sex combs. These animals died prior to full pigmentation of the abdomen, preventing evaluation of phenotypes observed in adult *mxc*^*G46*^ males [52]. Interactions between *CycG* and *crm* gave similar results. Eighty percent of *crm*^*7*^/Y flies presented sex combs on L2 and 35% on L3. Males combining *crm*^*7*^/Y and *CycG* loss of function died before the pupal stage, preventing analysis. In the few pharate escapers combining *crm*^*7*^/Y and *CycG* overexpression, leg transformations were significantly suppressed (Additional file 1).

**Figure 5:**
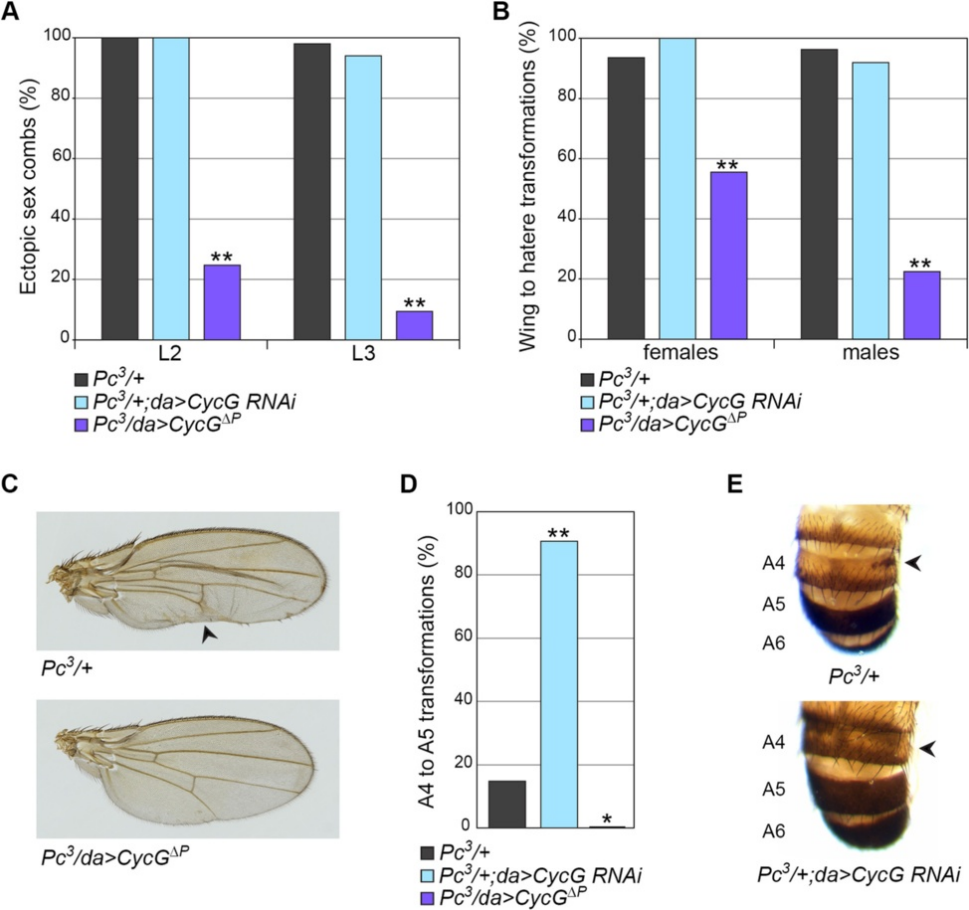
*CycG* misregulation alters homeotic transformations of a *Pc* mutant. **(A)** Penetrance of L2 and L3 ectopic sex combs in *Pc* ^*3*^/+, *Pc* ^*3*^/+;*da* > *CycG RNAi* and *Pc* ^*3*^/*da* > *CycG* ^*ΔP*^ males. Fisher’s exact test, **P < 0.0001 (*n* ≥ 58). **(B)** Penetrance of wing to haltere transformations in *Pc* ^*3*^/+, *Pc* ^*3*^/+;*da* > *CycG RNAi* and *Pc* ^*3*^/*da* > *CycG* ^*ΔP*^ females and males. Fisher’s exact test, **P < 0.0001 (*n* ≥ 31). **(C)** Representative *Pc* ^*3*^/+ adult female wing with posterior deformation corresponding to a partial transformation into haltere tissue (arrowhead). Representative *Pc* ^*3*^/*da* > *CycG* ^*ΔP*^ female wing with suppressed haltere to wing transformation. **(D)** Penetrance of A4 to A5 abdominal segment transformations in *Pc* ^*3*^/+, *Pc* ^*3*^/+;*da* > *CycG RNAi* and *Pc* ^*3*^/*da* > *CycG* ^*ΔP*^ males. Fisher’s exact test, *P < 0.05, **P < 0.0001 (*n* ≥ 29). **(E)** Abdomens of *Pc* ^*3*^ and *Pc* ^*3*^/+;*da* > *CycG RNAi* males with representative A4 to A5 transformations (arrowheads). A4, A5, A6: abdominal segments 4, 5, and 6.

Next, we tested several genes encoding members of PRC2. Neither *E*(*z*)^*63*^/+ nor *esc*^*21*^/+ males presented ectopic sex combs, and this was not significantly modified by *CycG* misregulation. On the other hand, mutants for *polyhomeotic* (*ph*) and *Polycomb* (*Pc*), encoding PRC1 subunits, both present ectopic sex combs. In *ph*-*p*^*410*^/Y males, 100% of L2 and L3 carried combs, and these phenotypes were significantly suppressed by *CycG* gain of function. Furthermore, the 20% of L2 and 5% of L3 sex combs of *ph*^*220*^/Y males were totally suppressed by *CycG* gain of function, and significantly enhanced by *CycG* loss of function (see Additional file 1). Finally, *Pc*^*3*^/+ males exhibit sex combs on 100% of L2 and 98% of L3 which were drastically suppressed by *CycG* gain of function (Figure 5A).

We also examined other *PcG*-induced homeotic phenotypes, focusing first on wing to haltere transformations. Ninety-three percent of female and 96% of male *Pc*^*3*^/+ wings exhibit a partial wing into haltere transformation of the area close to the posterior wing, inducing deformation of the wing (Figure 5B,C). *CycG* loss of function had no effect on this *Pc*^*3*^ phenotype, whereas *CycG* gain of function reduced both its strength and its penetrance (Figure 5B,C).

Similar to *Asx*^*3*^/+ males, several *PcG* and *ETP* mutants present a partial transformation of A4 into A5. A few *esc*^*21*^/+ or *Dsp1*^*1*^/Y males presented this phenotype, which was not significantly affected by *CycG* misregulation (Additional file 1). However, 15% of *Pc*^*3*^/+ males presented such A4 transformations (Figure 5D,E), whose strength and penetrance were significantly enhanced by *CycG* loss of function and completely suppressed by *CycG* gain of function (Figure 5D,E). Ninety-seven percent of *ph*-*p*^*410*^ males exhibited A4 into A5 transformations that were significantly suppressed by *CycG* gain of function (Additional file 1). The effect of *CycG* loss of function was not analyzed in *ph*-*p*^*410*^ males because pharates died prior to abdominal pigmentation. In *ph*^*220*^/Y males, posterior A4 transformations were not modified by *CycG* loss of function but significantly suppressed by *CycG* gain of function (Additional file 1).

All these genetic data point toward a role of *CycG* similar to that of a *PcG* gene as *PcG* loss of function phenotypes were enhanced by *CycG* loss of function but suppressed by *CycG* gain of function.

### *CycG* gain of function enhances *trxG*-induced homeotic transformations

**Figure 6:**
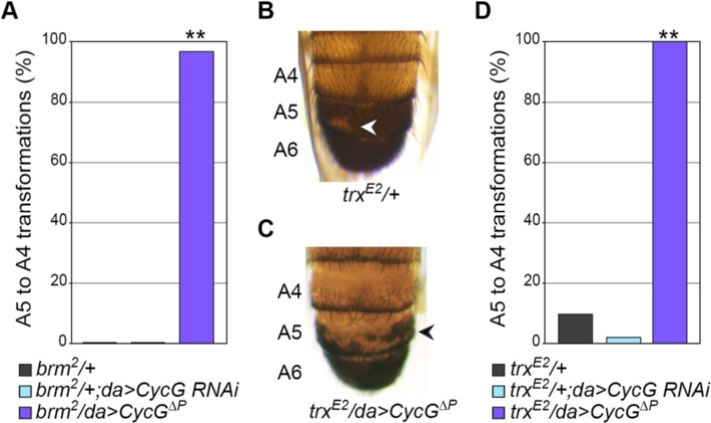
*CycG* gain of function enhances abdominal transformations of *trxG* mutants. **(A**, **D)** Penetrance of A5 to A4 transformations in adult male cuticles. Effect of *CycG* misregulation on *brm* ^*2*^-induced **(A)** or *trx* ^*E2*^-induced **(D)** A5 to A4 transformations. **(B**, **C)** Male abdominal cuticles. Representative *trx* ^*E2*^/+ **(B)** and *trx* ^*E2*^/*da* > *CycG* ^*ΔP*^ **(C)** males present, respectively, small and large light-pigmented cuticle patches on A5, denoting a partial transformation of abdominal segment A5 into A4 (white and black arrowheads). Fisher’s exact test, **P < 0.0001 (*n* ≥ 30). A4, A5, A6: abdominal segments 4, 5, and 6.

We next examined the effects of *CycG* misregulation on mutant phenotypes induced by loss of function of *trxG* genes. We studied mutations in *brm*, *ash1*, and *trx*, focusing on posterior abdominal pigmentation patterns, in particular A5 depigmentation indicating transformation of segment A5 into A4 [53]. Males *brm*^*2*^/+ did not present A5 toward A4 transformations, whereas 97% of males combining *brm*^*2*^/+ and *CycG* gain of function (*n* = 30) presented this phenotype (Figure 6A). *ash1*^*B1*^/+ males exhibited A5 to A4 transformations with a low penetrance (3%, *n* = 31). This phenotype was increased by *CycG* gain of function, although not to a significant level (13%, *n* = 24). Finally, 10% of *trx*^*E2*^/+ males presented partial transformation of A5 into A4 (Figure 6B) (*n* = 31). This transformation was decreased, though not significantly, by *CycG* loss of function (3%; *n* = 30), whereas both its strength and penetrance were drastically enhanced by *CycG* gain of function (100%, *n* = 30; Fisher’s exact test, *P* < 0.0001) (Figure 6C,D).

Together, these data show that *CycG* loss of function tended to decrease segmental identity transformations of *trxG* loss of function mutants, whereas *CycG* overexpression increased them. Hence, *CycG* antagonizes *trxG* genes in the posterior abdomen, behaving again as a *PcG* gene.

### *CycG* participates in *PcG*-dependent repression of *Scr* in leg imaginal discs

**Figure 7:**
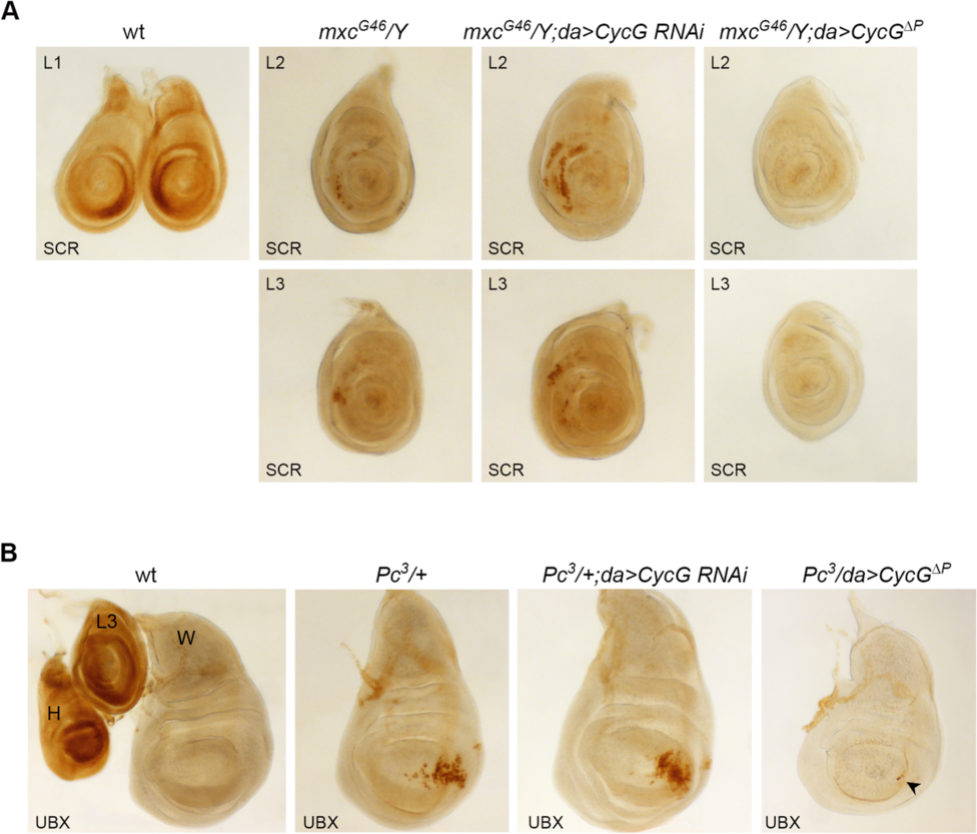
*CycG* misregulation modulates Hox protein profiles of *PcG* mutants. **(A)** Anti-SCR immunostainings of third instar larval leg imaginal discs from wild-type (wt), *mxc* ^*G46*^/Y, *mxc* ^*G46*^/Y;*da* > *CycG RNAi* or *mxc* ^*G46*^/Y;*da* > *CycG* ^*ΔP*^ males. **(B)** Anti-UBX immunostaining of third instar haltere (H), wing (W), and prothoracic leg (L3) imaginal discs from a wild-type female larva, and anti-UBX staining of wing discs from *Pc* ^*3*^/+, *Pc* ^*3*^/+;*da* > *CycG RNAi* or *Pc* ^*3*^/*da* > *CycG* ^*ΔP*^ females. Note the reduced ectopic staining in the *Pc* ^*3*^/*da* > *CycG* ^*ΔP*^ female wing disc (arrowhead).

Ectopic sex combs of *PcG* mutants are due to loss of silencing of the homeotic gene *Sex combs reduced* (*Scr*) in the second and third pairs of leg imaginal discs during larval development, leading to acquisition of a partial first-leg identity [54]. We therefore investigated the effect of *CycG* misregulation on expression of the *Scr* gene that specifies identity of the first thoracic segment in *Drosophila melanogaster* [55,56]. We monitored the pattern of SCR by immunostaining of leg imaginal discs, the larval structures that differentiate into legs during metamorphosis. In wild-type third instar larvae, cells with high levels of SCR form two semicircles in the L1 imaginal disc territory that gives rise to the anterior tibia and first tarsal segment (Figure 7A), whereas L2 and L3 imaginal discs present no SCR positive cells. Importantly, neither *CycG* loss of function nor *CycG* gain of function male larvae exhibited any detectable alteration in the spatial pattern of SCR (data not shown).

We next looked at SCR distribution in third instar leg discs from three different *PcG* mutant males (*mxc*^*G46*^, *Pc*^*3*^, *ph*-*p*^*410*^). We observed ectopic SCR in the anterior compartment of L2 and L3 imaginal discs in all three contexts (Figure 7A, Additional file 3). In male larvae combining *mxc*^*G46*^ and *CycG* loss of function, size of this ectopic SCR domain in both L2 and L3 discs was significantly increased (Figure 7A; Additional file 4). Conversely, in male larvae combining *mxc*^*G46*^ and *CycG* gain of function, the ectopic SCR domain was almost completely suppressed (Additional file 4). Ectopic SCR domains were also significantly enlarged in L2 and L3 discs of male larvae combining either *Pc*^*3*^ or *ph*-*p*^*410*^ with *CycG* loss of function and decreased in those combining *Pc*^*3*^ or *ph*-*p*^*410*^ with *CycG* gain of function (Additional files 3 and 4).

These results indicate that modulation of *PcG*-induced ectopic sex comb phenotypes by *CycG* reflects modulation of the SCR pattern in imaginal discs. Interestingly, *CycG* loss of function enlarged the SCR domain even in *ph*^*410*^ and *Pc*^*3*^ individuals who presented a quasi complete penetrance of the ectopic sex comb phenotype in adults. Together, these data strongly suggest that *CycG* cooperates with *PcG* genes in epigenetic *Scr* repression.

### *CycG* represses *Pc*^*3*^-induced ectopic Ultrabithorax in wing imaginal discs

*CycG* gain of function suppressed *Pc*^*3*^-induced wing to haltere transformations. This homeotic transformation is caused by ectopic expression of the homeotic gene *Ultrabithorax* (*Ubx*) that specifies haltere identity [57]. We therefore analyzed the effect of *CycG* misregulation on the UBX profile in wing imaginal discs. In wild-type third instar larvae, we observed a high level of UBX in haltere and third leg imaginal discs, whereas no UBX was detected in wing discs (Figure 7B). This pattern was not altered by *CycG* misregulation (data not shown). We observed patches of ectopic UBX in the posterior compartment of *Pc*^*3*^/+ wing imaginal discs (Figure 7B), that is, in the region that will be partly transformed into haltere in adults. *CycG* loss of function had no significant effect on this ectopic UBX profile, whereas *CycG* gain of function almost completely suppressed it (Figure 7B, Additional file 4).

These data show that the suppression of *Pc*^*3*^-induced wing to haltere transformation by *CycG* gain of function correlates with a suppression of ectopic UBX in wing imaginal discs, suggesting a role for *CycG* in *PcG*-dependent repression of *Ubx* in this tissue.

## Discussion

*Drosophila* Cyclin G participates in control of the cell cycle and in transcriptional regulation [39,45,46,58]. Physical interaction between Cyclin G and the ETP Corto suggests that Cyclin G might be related to *PcG* and *trxG* genes involved in maintenance of gene silencing and gene activation, respectively. The present work strengthens this hypothesis, since we demonstrate that Cyclin G directly binds the ETP ASX as well, co-localizes extensively with ASX on chromatin, and genetically interacts with *Asx*. Furthermore, Cyclin G mostly binds open chromatin in which genes are undergoing transcription. We also addressed the connection between Cyclin G and the PcG/TrxG system by investigating genetic interactions with a broad range of *PcG* and *trxG* alleles. We describe strong interactions between *CycG* misregulation and several *PcG* and *trxG* genes, together with modification of ectopic Hox protein expression induced by *PcG* mutations. Our results link *CycG* to maintenance of *Hox* gene expression patterns during development, and sustain a role for Cyclin G in epigenetic maintenance of transcription, a mechanism that is essential for transmission of gene expression patterns to daughter cells.

### Cyclin G, a modulator of Enhancers of Polycomb and Trithorax activity?

*Drosophila* Cyclin G was shown previously to physically interact with the ETP Corto [39]. Here, we demonstrate that this cyclin also binds the ETP ASX. Unlike Corto, which to date has only been detected in arthropods, ASX proteins are conserved in mammals. These proteins exhibit two remarkable domains, the ASX homology domain (ASXH), an amino terminal region containing two consensus sequences for nuclear receptor binding needed for ASX binding to the repressive PR-DUB complex [28], and a PHD domain located at the carboxy-terminal end that interacts with DNA. Mouse and human ASXL1 also contain a number of cyclin recognition sites and motifs for phosphorylation by CDKs scattered along the protein, suggesting that they are phosphorylated by a Cyclin/CDK dimer [59]. Indeed, the presence of a cyclin binding motif close to a CDK target sequence is essential for optimal phosphorylation of cyclin/CDK targets [60]. We show here that CycG binds the C-terminal region of ASX (amino-acids 1139 to 1668), a region which contains a cyclin interaction domain (amino-acids 1210 to 1214) as well as a substrate motif for phosphorylation by CDKs (amino-acids 1261 to 1267) [61]. Hence, a Cyclin G/CDK complex might phosphorylate ASX and modulate its activity. Nuclear Corto is highly phosphorylated [62] and although Corto lacks a canonical cyclin recognition motif, it contains a substrate motif for phosphorylation by CDKs in the Cyclin G interacting region [39]. Interestingly, this CDK target sequence is located in the chromodomain, involved in chromatin binding [63], suggesting that association of Corto with chromatin might be regulated by phosphorylation by a Cyclin G/CDK complex.

Phosphorylations are of paramount importance to regulate PcG and TrxG protein activity through the cell cycle. For example, in mammals, binding of the PcG protein BMI1 to chromatin is cell-cycle regulated and correlates with its phosphorylation status [64]. Furthermore, phosphorylation of PcG proteins EZH2 and SCML2 by cyclin/CDK complexes is regulated through the cell cycle [65,66]. These findings highlight a direct crosstalk between the Polycomb system of cellular memory and the cell-cycle machinery in mammals. Interestingly, fly Cyclin G controls the G1/S phase transition of the cell cycle and interacts with several CDKs (that is, CDK1, 2, 4, 5) [45,46,67,68]. Further investigations will be needed to determine whether a Cyclin G/CDK dimer phosphorylates the ETPs ASX and Corto and modulates their activity during the cell cycle.

### *Cyclin G* interacts with *ETP*, *PcG*, and *trxG* genes in the regulation of homeotic genes

ASX is involved in both activation and repression of homeotic genes [32], and this role is conserved by its mammalian homologs ASXL1 and ASXL2 [33,34]. Accordingly, some *Asx* mutants present both *PcG*-like and *trxG*-like transformations. For example, male *Asx*^*P1*^ flies bear partial transformation of abdominal segment A4 into A5, revealed by patches of dark pigmentation on A4, as well as partial transformation of A5 into A4, revealed by patches of unpigmented cuticle into A5 [69]. In a mouse *Asxl2* mutant, vertebras present both posterior and anterior transformations corresponding to *PcG* and *trxG* phenotypes, respectively [33]. We show here that deregulation of *CycG* impacts on homeotic phenotypes of an *Asx* mutant that presents only *PcG* phenotypes, that is, ectopic sex combs and transformation of A4 into A5. Importantly, neither inactivation nor overexpression of *CycG* induces *PcG* or *trxG* phenotypes *per se*. Nevertheless, *CycG* inactivation enhances ectopic sex combs induced by *Asx*, while *CycG* overexpression suppresses both *PcG*-like phenotypes. Combining the *Asx* mutation with *CycG* overexpression even leads to a *trxG*-like transformation of A5 into A4. In agreement with these results, homeotic phenotypes of mutants for *PcG* genes *crm*, *mxc*, *Pc*, and *ph* are all enhanced by *CycG* inactivation and suppressed by *CycG* overexpression. On the other hand, transformation of abdominal segment A5 into A4 observed in *trxG* mutants was enhanced (*ash1*, *trx*) and even induced (*brm*) by *CycG* overexpression. Hence, in both posterior legs and abdominal segments A4 and A5, *CycG* behaves as an enhancer of *PcG* genes.

*CycG* overexpression, as well as *CycG* inactivation, induce developmental delay (unpublished data) and flies overexpressing *CycG* suffer from the *Minute* syndrome [45,46]. In *Drosophila melanogaster*, the *Minute* syndrome has been described as a dominant, haploinsufficient phenotype that includes delayed development, short and thin bristles together with poor fertility and longevity [70]. More than 50 *Minute* loci were genetically identified; 15 of them were characterized molecularly and contain genes encoding ribosomal proteins [71]. Genetic screens designed to isolate new *PcG* and *trxG* genes in flies have frequently identified *Minute* mutants as suppressors of *PcG* mutations [19,72]. This effect has been considered to be unspecific, since other factors resulting in developmental delays, that is, low temperature, also suppress the ectopic sex comb phenotype of *PcG* mutants [73]. Although homeotic phenotypes of *PcG* mutants are suppressed by *CycG* overexpression, the fact that *CycG* inactivation associates developmental delay and enhancement of *PcG* transformations indicates that *CycG* acts as a specific modifier of these phenotypes. Hence, our data characterize *CycG* as a *bona fide* enhancer of *Polycomb*-group genes, involved in *Hox* gene regulation.

### Cyclin G, a transcriptional activator or repressor?

In agreement with our genetic analyses, we observed that *CycG* inactivation enlarges ectopic SCR domains in *PcG* mutant leg imaginal discs, whereas *CycG* overexpression eliminates ectopic SCR and UBX domains in leg and wing imaginal discs. This suggests that Cyclin G facilitates the maintenance of *Scr* and *Ubx* silencing by PcG complexes in these imaginal tissues. Nevertheless, whether this effect is direct or not remains to be determined. Paradoxically, we previously observed that Cyclin G is required for maintenance of *Abd*-*B* expression in the epithelium of abdominal segments A5 and A6 in young female pupae [58]. This effect might be direct as Cyclin G was shown to bind the *Abd*-*B* promoter and the *iab*-*7* polycomb response element in embryos [39]. Altogether, these data show that Cyclin G is involved in epigenetic regulation of *Hox* gene expression, acting as a repressor or an activator depending on the tissue, the developmental stage, and the target gene. *CycG* is thus similar to many other genes encoding maintenance proteins that affect transcription differently depending on the context. For example, *Drosophila E*(*z*) is classified as a *PcG* repressor, but behaves genetically as an *ETP* [17,19]. Furthermore, *brahma* (*brm*) as well as most genes encoding members of the SWI/SNF chromatin remodeling complex Brahma-associated protein (BAP) are classified as *trxG* activators, but *snr1*, that encodes a conserved subunit of BAP, participates in cell-type specific transcriptional repression in the developing *Drosophila* wing [74].

To tackle the molecular mechanisms by which Cyclin G controls transcription, we analyzed its binding to polytene chromosomes in larval salivary glands. Cyclin G binds DAPI interbands indicative of open chromatin, co-localizes largely with RNA Pol II CTD phosphorylated on serine 2, and shows very few overlaps with H3K27me3. All these data point to the presence of Cyclin G on actively transcribed genes. Deregulation of *CycG* does not induce homeotic transformations *per se*, but modulates those due to *PcG* or *trxG* mutations. Cyclin G might thus preferentially affect ‘destabilized’ genes. Since Cyclin G co-localizes with DAPI interbands and with RNA Pol II phosphorylated on serine 2, and behaves as a *PcG* enhancer, its role could possibly be to moderate the expression of active genes.

We also show that Cyclin G extensively overlaps with ASX. As Cyclin G and ASX co-immunoprecipitate, Cyclin G and ASX can be assumed to interact on chromatin. ASX belongs to the repressive PR-DUB complex, which contains the histone deubiquitinase BAP1, also called Calypso in *Drosophila* [28]. PRC1 ubiquitinates H2A on lysine 119, and this ubiquitin residue can be removed by PR-DUB. Surprisingly, disruption of PR-DUB enzymatic function led to impaired *Hox* gene repression, as does a shortage of PRC1. Hence, PcG silencing has been proposed to depend on a dynamic equilibrium between H2A ubiquitination by PRC1 and deubiquitination by PR-DUB [28]. An interesting possibility could then be that Cyclin G influences this equilibrium by modulating ASX activity.

In embryonic stem cells, some key developmental genes, called bivalent, are simultaneously stamped by both repressive (H3K27me3) and activating (H3K4me3) histone marks [75]. Although these genes are associated with RNA Pol II CTD phosphorylated on serine 5, they are transcribed at a low level [76]. Ubiquitination of H2A by PRC1 controls this process, and has therefore been suggested to control transcriptional elongation [76,77]. These data raise the exciting possibility that Cyclin G fine-tunes transcription by controlling H2A ubiquitination *via* interaction with the ASX subunit of PR-DUB.

## Conclusions

Our findings highlight a crosstalk between the Polycomb system and *Drosophila* Cyclin G. The importance and complexity of the interaction between Cyclin G and ASX warrant further investigation. It is tempting to speculate that this interaction regulates transcriptional elongation. Specific points to be explored in the near future include interaction between Cyclin G and ASX in the context of PR-DUB, involvement of Cyclin G in the balance between ubiquitinated and unubiquitinated H2A, and regulation of PR-DUB activity by Cyclin G through the cell cycle.

## Methods

### *Drosophila* strains and genetic analyses

*Drosophila melanogaster* stocks were raised on standard yeast-cornmeal medium at 25°C. Transgenic lines *UAS*::*dsCycG2* (referred to as *CycG RNAi*) [39] and *UAS*::*CycG*^*ΔP*^ (line *RCG76*, allowing expression of Cyclin G deleted of the 25 C-terminal amino-acids, containing a putative PEST sequence [45,46]) were used to inactivate *CycG* by RNA interference (*CycG* loss of function) or overexpress *CycG* (*CycG* gain of function), respectively. *CycG* misregulations were induced using the ubiquitous driver *daughterless* (*da*::*Gal4*). The third chromosome transgenes *da*::*Gal4* and *UAS*::*CycG*^*ΔP*^ were recombined and gave rise to chromosome *da*::*Gal4*,*UAS*::*CycG*^*ΔP*^, called *da* > *CycG*^*ΔP*^. Chromosome *da* > *CycG*^*ΔP*^ was maintained in males at 18°C to overcome female sterility and high lethality associated with *CycG* overexpression at 25°C [45]. For co-immunoprecipitations, a new transgenic line containing a *UAS*::*Myc*-*CycG*^*ΔP*^ construct was obtained by PhiC31 integrase-mediated insertion of *pUASP*-*Myc*-*CycG*^*ΔP*^-*attB* at 51C (stock BL-24482) [78].

Alleles of *PcG*, *trxG*, or *ETP* genes used in this study are listed in Additional file 2, and their characteristics are described in [79]. Genetic interactions between these genes and *CycG* were assessed in *trans*-heterozygous flies obtained by crossing females heterozygous for a balanced *PcG*, *trxG*, or *ETP* mutation with males either *da* > *CycG RNAi*, *da* > *CycG*^*ΔP*^, or *da*::*Gal4* as a control. All crosses were performed at 25°C, and parents were transferred to new vials every 3 days. Penetrance of homeotic phenotypes affecting legs, wings, or abdomen was determined among the progeny. Phenotypes were assessed by examining 30 flies for each genotype (whenever possible) under a dissecting microscope. Wings and male legs were mounted in Hoyer’s medium. Sex combs were counted under a microscope at × 100 magnification. Statistical significance of results was evaluated using *t*-test and Fisher’s exact test on GraphPad QuickCalcs Web site: http://www.graphpad.com/quickcalcs/contingency1/ (accessed April 2014).

### Plasmid constructs

The 3′ sequence of *Asx* (bp 3415 to stop codon, Dmel_CG8787) was amplified from embryonic cDNAs using primers *AsxC*_F (5′-caccgccgccatgacgcgtcctgccaatgcatcacc-3′) and *AsxC*_R (5′-tcatcatctaatcacacaggcgacacacagc-3′). The full-length *CycG* cDNA was amplified using primers *CycGnF* 5′-cacctctgtccctgtacgctactcc-3′ and *CycGnR* 5′-ctaacattgttcgaaaattggaattatggg-3′. cDNAs encoding truncated forms of Cyclin G (Cyclin G 1 to 130 and Cyclin G 130 to 566) were amplified using primers *CycGnF* and *CycG1*-*130R* 5′-ctaggcagcctgggccgaagtcgagggctg-3′, and *CycG130*-*566 F* 5′-caccgccgctgctgccgcatcc-3′ and *CycGnR*, respectively. PCR products were cloned into pENTR/D-TOPO® (Invitrogen), then transferred into Gateway® vector *pAMW* (Invitrogen, a gift from T. Murphy) to produce the Myc-ASX-C fusion protein under control of the *actin5C* promoter, or *pAFW* (Gateway®, Invitrogen, Carlsbad, CA, USA) to produce FLAG-tagged Cyclin G fusion proteins under control of the same promoter.

### Cell transfection and protein-protein interactions

The yeast two-hybrid screen performed to find interactors of the ASX-C terminal domain (ASX-C, residues 1139 to 1668) has been described previously [48,80]. *Drosophila* S2 cells were cultivated at 25°C in Schneider’s *Drosophila* medium supplemented with 10% fetal calf serum and antibiotics. Then 5.10^6^ cells were transfected into 25 cm^2^ flasks using Effecten® Transfection reagent kit at a 1/10 DNA-Effecten® ratio according to the manufacturer’s instructions (Effecten®; QIAGEN, Venlo, Limburg, The Netherlands). Cells were collected 36 or 72 h after transfection depending on the constructs, and total protein extracts were prepared as previously described [45]. Cross-linking was performed by treatment of cells with 1% paraformaldehyde for 10 min on ice prior to protein extraction. For co-immunoprecipitation, 500 μg of total cell extracts were incubated overnight at 4°C with 3 μg of either goat anti-Myc antibody (ab9132, Abcam, Cambridge, UK), mouse monoclonal anti-FLAG antibody (F3165, Sigma, St. Louis, MO, USA), or goat anti-HA as mock antibody (sc-805, Santa Cruz Biotechnology, Santa Cruz, CA, USA). Then, 30 μl of Bio-Adembeads Protein G (Ademtech, Westbury, New York, USA) were then incubated with the cell lysate for 3 h at 4°C. The beads were washed three times in ELB buffer [45] and resuspended in 30 μl of the same buffer. Furthermore, 20 μl of input, 20 μl of supernatant, and half of the beads were used for Western analysis. Immunoprecipitates were detected with rabbit polyclonal anti-Myc antibody (1:5,000; A00172, GenScript) and mouse monoclonal anti-FLAG antibody (1:2,000).

For co-immunoprecipitation in larvae, protein extracts were prepared from *da* > *Myc*-*CycG*^*∆P*^ third instar larvae, previously cleared of gut and fat body and treated with 1% paraformaldehyde for 10 min at room temperature. Five micrograms of either goat anti-Myc antibody, sheep anti-ASX N-ter antibody (described in [81]), or goat anti-HA as mock antibody were incubated with 50 μl of Dynabeads® Protein G (Life Technologies, Carlsbad, CA, USA) for 3 h at 4°C. The bead-antibody complexes were washed in ELB buffer and incubated with 1 mg of protein extracts overnight at 4°C. The beads were washed three times in ELB buffer before Western analysis. Immunoprecipitates were detected with goat anti-Myc antibody (1:5,000).

### Immunolocalization on polytene chromosomes

Squashes of *w*^*1118*^ third instar larval salivary glands and immunostainings were performed as described previously [36] using guinea pig anti-Cyclin G (1:40), sheep anti-ASX N-ter (1:20) (described in [39] and [81], respectively), rabbit anti-H3K27me3 (1:40; pAb-069-050, Diagenode, Denville, NJ, USA), or rabbit anti-RNA polymerase II CTD phosphorylated on serine 2 (1:200; ab5095, Abcam, Cambridge, UK) antibodies. Secondary antibodies (Alexa Fluor® 488 goat anti-guinea pig, Alexa Fluor® 594 goat anti-rabbit IgG and Alexa Fluor® 680 donkey anti-sheep IgG, Molecular Probes, Eugene, OR, USA) were used at a 1:1,000 dilution.

### Immunostaining of imaginal discs

For each genotype, at least 15 third instar wandering larvae were dissected and fixed in 3.7% paraformaldehyde for 20 min at room temperature, then immunostained according to [82,83] using rat polyclonal anti-SCR antibody (1:100 [82]) or mouse anti-UBX monoclonal antibody (1:20; FP3.38 [84]). The universal biotinylated antibody (Vector Laboratories, CA, USA) was used at a 1:200 dilution. Staining was performed with VECTASTAIN Elite ABC system (Vector Laboratories, CA, USA) using DAB as substrate (D4418, Sigma, St. Louis, MO, USA). Note that for a given antibody, discs of all genotypes were incubated for the same length of time in DAB. Imaginal discs were mounted in PBS:glycerol (50:50). All pictures were acquired with a QICAM Fast 1394 digital camera, at × 100 magnification. Staining was quantified by calculating the percentage of stained area in the discs using Image J. SCR positive area was measured as a percentage of the total leg disc area, and UBX positive area was evaluated relative to the presumptive wing blade and hinge area of the wing disc. Statistical significance of results was evaluated using *t*-test.

## Additional files

Additional file 1: Table S1. Effect of *CycG* misregulation on *PcG*-induced homeotic transformations. L1, L2, and L3: prothoracic, mesothoracic and metathoracic legs. A4 and A5: abdominal segments 4 and 5. Fisher’s exact test **P* < 0.05 ***P* < 0.0001. NA, not analyzed. ^a^ *n* = 81. Additional file 2: Table S2.
*trxG*, *PcG* and *ETP* alleles used in this study. These alleles are described in [79]. Additional file 3: Figure S1.
*CycG* misregulation modulates Hox protein profiles of *Pc* ^*3*^ and *ph*-*p* ^*410*^. Anti-SCR immunostainings of third instar larval leg imaginal discs from (A) *Pc* ^*3*^/+, *Pc* ^*3*^/+; *da* > *CycG RNAi*, and *Pc* ^*3*^/*da* > *CycG* ^*ΔP*^ males, and (B) *ph*-*p* ^*410*^/Y, *ph*-*p* ^*410*^/Y;*da* > *CycG RNAi* and *ph*-*p* ^*410*^/Y;*da* > *CycG* ^*ΔP*^ males. L2, L3: mesothoracic and metathoracic leg imaginal discs. H, haltere imaginal disc. Additional file 4: Table S3. Size of ectopic SCR and UBX domains in imaginal discs of *PcG* mutants. n: number of imaginal discs analyzed. L2, L3: mesothoracic and metathoracic leg imaginal discs. *t*-test **P* < 0.05, ***P* < 0.005, and ****P* < 0.0005. NA, not analyzed.

## Abbreviations

A4: A5, A6, abdominal segment 4, 5, 6
Abd-B: abdominal B
ASH1: absent, small, or homeotic discs 1
ASX: Additional sex combs
BRM: Brahma
CCNG1: Cyclin G1
CCNG2: Cyclin G2
CDK: cyclin-dependent kinase
crm: cramped
DSP1: dorsal switch protein 1
ESC: extra sex comb
ETP: enhancer of trithorax and polycomb
E(Z): enhancer of zeste
H3K27me3: tri-methylated histone H3 lysine 27
L1: prothoracic leg
L2: mesothoracic leg
L3: metathoracic leg
mxc: multi sex combs
PC: polycomb
PcG: polycomb-group
PH: polyhomeotic
PR-DUB: polycomb repressive deubiquitinase
PRC: polycomb repressive complex
PRE: Polycomb Response Element
RNA Pol II CTD: C-terminal domain of RNA polymerase II
Scr: Sex combs reduced
TAC1: trithorax activating complex 1
TrxG: trithorax-group
Ubx: *Ultrabithorax*
