# Supplementary material for: Drosophila Cyclin G and epigenetic maintenance of gene expression during development
Source: Epigenetics Chromatin. 2015 May 7;8:18. doi: 10.1186/s13072-015-0008-6 (PMC4438588; doi:10.1186/s13072-015-0008-6)
Supplement: Additional file 3: Figure S1. — CycG misregulation modulates Hox protein profiles of Pc 3 and ph-p 410. Anti-SCR immunostainings of third instar larval leg imaginal discs from (A) Pc 3/+, Pc 3/+; da > CycG RNAi, and Pc 3/da > CycG ΔP males, and (B) ph-p 410/Y, ph-p 410/Y;da > CycG RNAi and ph-p 410/Y;da > CycG ΔP males. L2, L3: mesothoracic and metathoracic leg imaginal discs. H, haltere imaginal disc. [file 13072_2015_8_MOESM3_ESM.doc]

**Figure S1. *CycG* misregulation modulates Hox protein profiles of *Pc^3^* and *ph-p^410^*.**


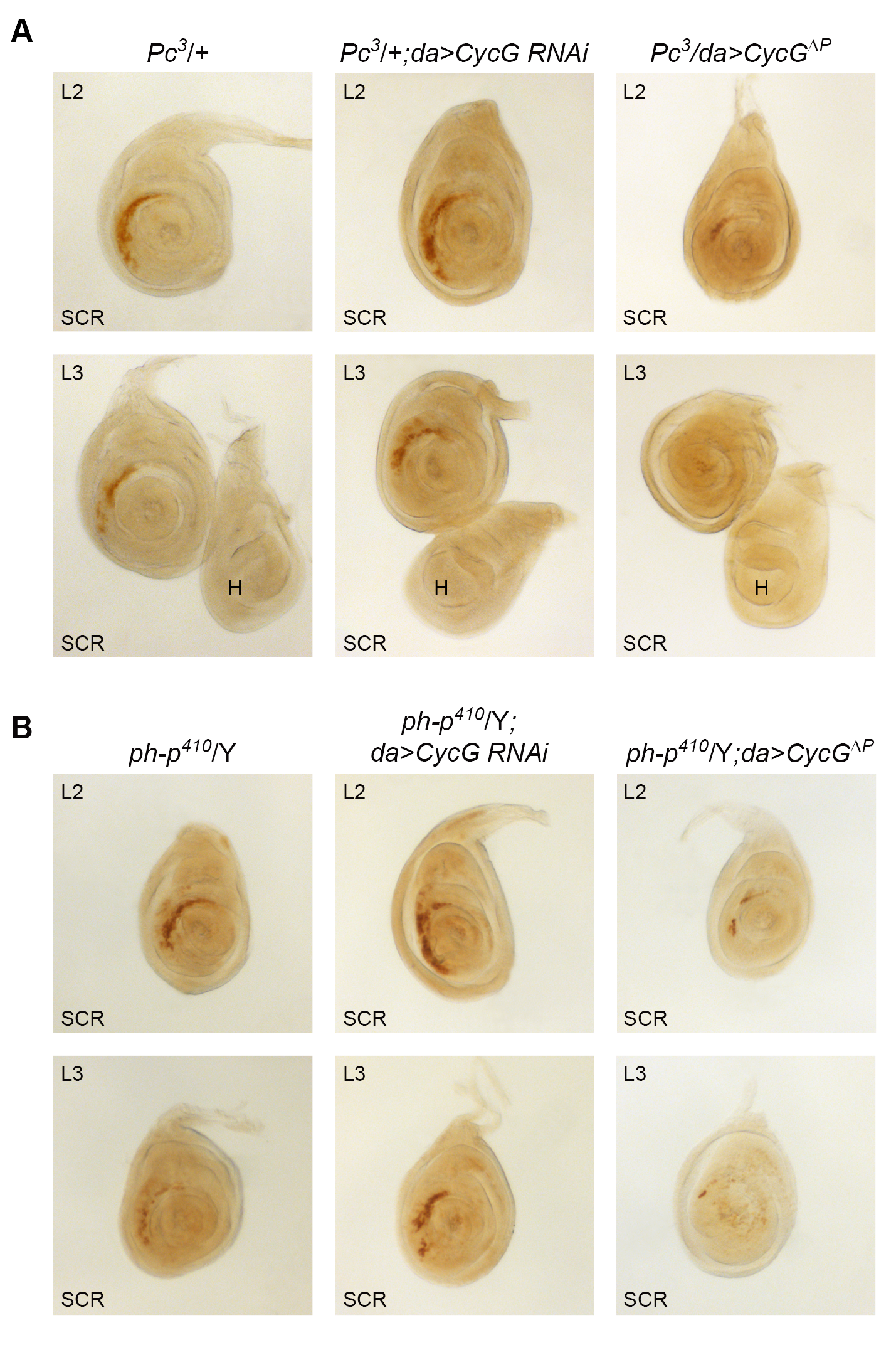


Anti-SCR immunostainings of third instar larval leg imaginal discs from (A) *Pc^3^*/+, *Pc^3^*/+;*da>CycG RNAi,* and *Pc^3^*/*da>CycG^ΔP^* males, and (B) *ph-p^410^*/Y*, ph-p^410^*/Y*;da>CycG RNAi* and *ph-p^410^*/Y*;da>CycG^ΔP^* males. L2, L3: mesothoracic and metathoracic leg imaginal discs. H: haltere imaginal disc.
